# Survival of Patients with Deficient Mismatch Repair Versus Proficient Mismatch Repair Metastatic Colorectal Cancer Receiving Curative-Intent Local Treatment of Metastases in a Nationwide Cohort

**DOI:** 10.1245/s10434-023-13974-7

**Published:** 2023-08-01

**Authors:** Koen Zwart, Frederieke H. van der Baan, Cornelis J. A. Punt, G. Emerens Wensink, Karen Bolhuis, Miangela M. Laclé, Wilhelmina M. U. van Grevenstein, Jeroen Hagendoorn, Ignace H. de Hingh, Miriam Koopman, Geraldine Vink, Jeanine Roodhart

**Affiliations:** 1 Department of Medical Oncology, University Medical Center Utrecht, Utrecht University, Utrecht, The Netherlands; 2 Department of Epidemiology, Julius Center for Health Sciences and Primary Care, University Medical Center Utrecht, Utrecht, The Netherlands; 3 Department of Medical Oncology, Amsterdam University Medical Center, University of Amsterdam, Amsterdam, The Netherlands; 4 Department of Medical Oncology, Netherlands Cancer Institute, Amsterdam, The Netherlands; 5 Department of Pathology, University Medical Center Utrecht, Utrecht University, Utrecht, The Netherlands; 6 Department of Surgery, University Medical Center Utrecht, Utrecht University, Utrecht, The Netherlands; 7 Department of Surgery, Catharina Hospital, Eindhoven, The Netherlands; 8 Department of Research and Development, Netherlands Comprehensive Cancer Organisation (IKNL), Utrecht, The Netherlands

## Abstract

**Background:**

It is unclear whether curative-intent local therapy of metastases is of similar benefit for the biological distinct subgroup of patients with deficient mismatch repair (dMMR) metastatic colorectal cancer (mCRC) compared with proficient mismatch repair (pMMR) mCRC.

**Patients and Methods:**

In this nationwide study, recurrence-free (RFS) and overall survival (OS) were analyzed in patients with dMMR versus pMMR mCRC who underwent curative-intent local treatment of metastases between 2015 and 2018. Subgroup analyses were performed for resection of colorectal liver metastases (CRLM) and cytoreductive surgery ± hyperthermic intraperitoneal chemotherapy (CRS ± HIPEC). Multivariable regression was conducted.

**Results:**

Median RFS was 11.1 months [95% confidence interval (CI) 8.5–41.1 months] for patients with dMMR tumors compared with 8.9 months (95% CI 8.1–9.8 months) for pMMR tumors. Two-year RFS was higher in patients with dMMR versus pMMR (43% vs. 21%). Results were similar within subgroups of local treatment (CRLM and CRS ± HIPEC). Characteristics differed significantly between patients with dMMR and pMMR mCRC; however, multivariable analysis continued to demonstrate dMMR as independent factor for improved RFS [hazard ratio (HR): 0.57, 95% CI 0.38–0.87]. Median OS was 33.3 months for dMMR mCRC compared with 43.5 months for pMMR mCRC, mainly due to poor survival of patients with dMMR in cases of recurrence in the preimmunotherapy era.

**Conclusion:**

Patients with dMMR eligible for curative-intent local treatment of metastases showed a comparable to more favorable RFS compared with patients with pMMR, with a clinically relevant proportion of patients remaining free of recurrence. This supports local treatment as a valuable treatment option in patients with dMMR mCRC and can aid in shared decision-making regarding upfront local therapy versus immunotherapy.

**Supplementary Information:**

The online version contains supplementary material available at 10.1245/s10434-023-13974-7.

Metastatic colorectal cancer (mCRC) is a highly heterogeneous disease with approximately 5% of patients having a tumor harboring deficient mismatch repair (dMMR).^1,2^ dMMR mCRC forms a poorer biological subgroup with distinct prognostic, predictive, and therapeutic implications compared with proficient mismatch repair (pMMR) mCRC.^2^ Local treatment of metastases prolongs overall survival (OS) and is, up to now, recommended in all patients with resectable mCRC.^3^ Consensus of curative-intent local therapy of metastases was mainly based on studies including patients with pMMR mCRC, and its benefit is not known for patients with dMMR mCRC.^3^ However, there are important differences between dMMR and pMMR tumors. Patients with dMMR mCRC obtain a lower response rate to palliative systemic therapy (5% vs. 44%)^4,5^ and the OS in the preimmunotherapy era is shorter (16 vs. 24 months).^6^ Furthermore, there are important differences in patterns of metastatic spread; dMMR tumors more often metastasize to the lymph nodes and pMMR tumors to the liver, making patients with dMMR tumors less often amenable to surgery (10% vs. 26%).^7^

Currently, immunotherapy leads to durable responses as a first-line systemic treatment in patients with dMMR mCRC with a prolonged progression-free survival of 16.5 months in patients receiving monoimmunotherapy compared with 8.2 months in patients receiving systemic palliative therapy in the randomized phase III KEYNOTE-177 trial.^8^ Around 30% of patients that received immunotherapy had primary progression; however, patients that responded to immunotherapy often had a long duration of response with 48% of patients being progression-free at 2 years. Furthermore, small studies have showed that salvage local treatment after immunotherapy treatment in patients with an irresectable primary tumor or mCRC is effective with a 2-year recurrence-free survival (RFS) of 62–81%, although complete histological responses were already observed in 56–93% of cases.^9–11^ This setting concerns salvage local treatment after immunotherapy. With the remarkable results of immunotherapy, the value of upfront local therapy treatment in patients with dMMR mCRC is questioned.^8^ However, there is only limited data regarding local treatment in patients with mCRC and resectable metastases without upfront immunotherapy.

The aim of this study is to describe the characteristics, RFS, and OS after curative-intent local treatment of metastases in patients with dMMR versus pMMR tumors in the period before immunotherapy, which was approved as standard of care. This allows evaluation of clinical outcomes after local treatment for patients with dMMR mCRC without upfront immunotherapy and will provide valuable knowledge for shared decision making regarding immunotherapy or upfront local treatment in case of resectable metastases.

## Patients and Methods

### Study Design and Data Collection

This is an observational nationwide study using specified individual patient data of The Netherlands Cancer Registry (NCR), collected from medical records of all Dutch hospitals by trained data managers. Race/ethnicity is not registered by the NCR, because this is not allowed in The Netherlands. All patients in The Netherlands receiving curative-intent local treatment of metastases with dMMR mCRC from 2015 to 2018 were selected, as well as those with pMMR mCRC from 2015 to 2016. Curative-intent local treatment of metastases included metastasectomy, radiofrequency ablation, or microwave ablation of all visible metastases.

MMR and/or microsatellite instability (MSI) status was collected from patient records when determined during routine clinical practice. Patients with unknown MMR and/or microsatellite instability status were excluded. MMR expression was defined as deficient when there was a loss in protein expression of either MLH1, PMS2, MSH2, or MSH6. MSI was assessed with the mononucleotide repeat pentaplex panel (BAT-25, BAT-26, NR-21, MONO-27, and NR-24) and determined as MSI high when at least two markers showed MSI. Identification of Lynch syndrome or sporadic dMMR/MSI-H was based on a tailored approach by MMR protein expression, family history, *BRAF*^*V600E*^ status, and *MLH1* promotor hypermethylation status, as previously described by Parsons et al.^12^ The NCR was linked with The Dutch Nationwide Pathology Databank (PALGA) to obtain original pathology reports, including *BRAF*^*V600E*^ and *RAS* status, if determined during routine clinical practice. Since *RAS* and *BRAF*^*V600E*^ mutations are considered mutually exclusive, *RAS* was considered wild-type when *RAS* mutation status was unknown and a *BRAF*^*V600E*^ mutation was present and vice versa.^13^

Local therapy was categorized into colorectal liver metastasectomy (CRLM), cytoreductive surgery with or without hyperthermic intraperitioneal chemotherapy (CRS ± HIPEC), pulmonary metastasectomy, lymph node metastasectomy, and other/combination local therapy (e.g., cerebral metastasectomy or combination of CRLM and CRS ± HIPEC). CRLM included liver metastasectomy, radiofrequency ablation, and microwave ablation. Sidedness of the primary tumor was defined as right-sided comprising tumors from cecum up to the transverse colon and left-sided from splenic flexure up to sigmoid. World Health Organization (WHO) performance status was measured before the start of treatment. Staging was determined by pathological stage and, when necessary, complemented with clinical stage.

### Outcome

RFS was the primary outcome and defined as time from local treatment to recurrence of disease or death, whichever occurred first, and OS as time from local treatment to death or last follow-up alive.

The NCR obtained information on RFS until August 2020 and was linked with the National Municipal Personal Records Database in February 2021 to obtain the most recent information on vital status. Imaging modalities and frequencies were performed according to local clinical practice. The NCR data were pseudomized and consent was obtained by an opt-out approach.

### Statistical Analysis

Differences between baseline characteristics were analyzed with a *t*-test for continous variables and chi-squared test for categorical variables. Kaplan–Meier curves were obtained with censoring of patients that were lost to follow-up. Median follow-up was calculated by the reverse Kaplan–Meier approach. Multiple imputation by a substantive model compatible with fully conditional specification was used for missing data.^14^ A univariable and multivariable Cox regression model was derived with ten preselected factors, based on literature and expert opinion.^15,16^ Proportional hazards were visually checked with Schoenfeld residuals and statistically tested. A *p*-value < 0.05 was considered statistically significant. All analyses were performed in R version 3.5.1 (packages ‘gtsummary’, ‘smcfcs’, ‘survminer’, ‘survival’, ‘prodlim,’ and ‘table’ were used).^17^

## Results

### Patient Characteristics

A total of 84 of 380 patients with dMMR mCRC (22%) received local treatment versus 1099 of 2319 patients with pMMR mCRC (47%), as expected based on the known different metastatic patterns and consequent lower amenability for local therapy for patients with dMMR mCRC. The mean age and WHO performance status was comparable between patients with dMMR mCRC and pMMR mCRC, while sex (38% vs. 58% male, *p*-value < 0.01), *BRAF*^*V600E*^ mutation status (50% vs. 4%, *p*-value < 0.01), *RAS* mutation status (21% vs. 52%, *p*-value < 0.01), site of primary tumor (78% right-sided vs. 27%, *p*-value < 0.01), liver-only disease (27% vs. 70%, *p*-value < 0.01), and peritoneal involvement (44% vs. 18%, *p*-value < 0.01) differed significantly (Table 1). This also led to significant differences in type of local therapy, whereas CRLM-only resection was performed in 27% of patients with dMMR mCRC compared with 70% of pMMR mCRC and CRS/HIPEC in 48% and 16%, respectively. Neoadjuvant systemic chemotherapy was significantly more often administered in patients with pMMR tumors than dMMR tumors (42% vs. 17%, *p*-value < 0.01). All patients were included in the period before immunotherapy, which was approved as standard of care.

**Table 1:** Patient and treatment characteristics

	pMMR (*N* = 1099)	dMMR (*N* = 84)	*p*-value
Sex, no. of male	632 (58%)	32 (38%)	**< 0.01**
Age in years, mean (SD)	61.1 (10.2)	63.1 (11.7)	0.14
Sidedness			
Left-sided	443 (41%)	14 (17%)	**< 0.01**
Right-sided	293 (27%)	65 (78%)	
Rectosigmoid/rectum	356 (32%)	4 (5%)	
Missing	7	1	
Resection status of primary tumor			
No resection	57 (5%)	1 (1%)	0.17
Resection	1042 (95%)	83 (99%)	
Differentation grade			
Well	22 (2%)	0 (0%)	**< 0.01**
Moderate	885 (89%)	37 (54%)	
Poor	83 (8%)	31 (46%)	
Missing	109	16	
T stage			
T1–3	782 (72%)	39 (47%)	**< 0.01**
T4	301 (28%)	44 (53%)	
Missing	16	1	
N stage			
N0	344 (32%)	28 (33%)	0.83
N1/2	746 (68%)	56 (67%)	
Missing	9	0	
Stage			
Stage I–III	335 (30%)	37 (45%)	**0.01**
Stage IV	764 (70%)	46 (55%)	
Missing	0	1	
WHO performance status			
0	331 (68%)	17 (63%)	0.88
1+	159 (32%)	10 (37%)	
Missing	609	57	
Metastatic localization			
Liver only	769 (70%)	23 (27%)	**< 0.01**
Peritoneal involvement	201 (18%)	37 (44%)	
Other	129 (12%)	24 (29%)	
Number of metastatic sites			
1	878 (80%)	72 (86%)	0.41
2+	221 (20%)	12 (14%)	
Molecular status			
*BRAF*^*V600E*^ mutation	22 (4%)	26 (50%)	**< 0.01**
*RAS* mutation	322 (52%)	11 (21%)	
*BRAF*^*V600E*^ and *RAS* wildtype	274 (44%)	15 (29%)	
Missing	481	32	
Lynch syndrome status			
Probable Lynch	–	21 (31%)	–
Sporadic	–	46 (69%)	
Missing	–	17	
Local therapy			
CRLM only	769 (70%)	23 (27%)	**< 0.01**
CRS/HIPEC only	172 (16%)	40 (48%)	
Pulmonary metastasectomy only	38 (3%)	1 (1%)	
Lymph node resection only	19 (2%)	10 (12%)	
Other/combination*	101 (9%)	10 (12%)	
Systemic therapy			
Neoadjuvant systemic therapy	457 (42%)	14 (17%)	**< 0 .01**
Adjuvant systemic therapy after local therapy	101 (9%)	13 (15%)	
No perioperative systemic therapy	541 (49%)	57 (68%)	

Bold-values are considered significant (*p*-value < 0.05)

*CRLM* colorectal liver metastases, *CRS* cytoreductive surgery, *dMMR* deficient mismatch repair, *HIPEC* hyperthermic intraperitoneal chemotherapy, *mCRC* metastatic colorectal cancer, *pMMR* proficient mismatch repair, *SD* standard deviation, *WHO* World Health Organization

*Other local treatment: metastasectomy of other organs (e.g., cerebral metastasectomy) or combination treatment (e.g., CRLM and CRS/HIPEC)

### Recurrence-Free and Overall Survival

**Fig. 1:**
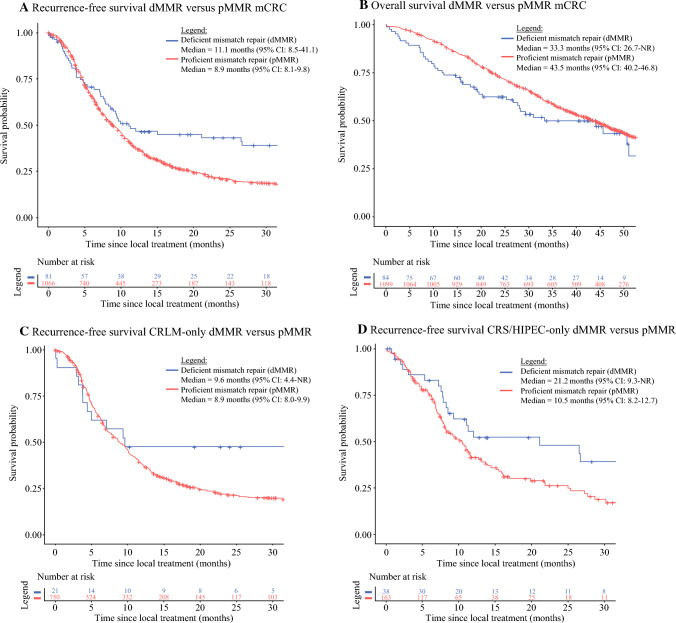
Recurrence-free and overall survival of deficient mismatch repair (dMMR) versus proficient mismatch repair (pMMR) metastatic colorectal cancer patients (**A**, **B**), recurrence-free survival in subgroups of colorectal liver metastases treated patients (**C**), and cytoreductive surgery/hyperthermic intraperitoneal chemotherapy treated patients (**D**)

Follow-up for patients with mCRC with a dMMR tumor was median 42.3 months [interquartile range (IQR) 29.5–49.1 months] and for pMMR it was a median of 51.6 months (IQR 45.8–58.6 months). Median RFS was 11.1 months (95% CI 8.5–41.1 months) for patients with a dMMR tumor compared with 8.9 months (95% CI 8.1–9.8 months) for pMMR. A proportion of 43% of patients with dMMR mCRC were recurrence-free 2 years after local treatment, compared with 21% of patients with pMMR. Stratified for type of local treatment, the same trend was shown for CRS ± HIPEC and resection of CRLM (Fig. 1). The improved RFS for patients with dMMR tumors is therefore not a result of differences in type of local therapy. No large differences in RFS were observed when stratifying for molecular groups. Patients with dMMR mCRC and a *BRAF*^*V600E*^-mutated tumor showed a median RFS of 7.1 months (95% CI 3.9–N/R months, *n* = 26), with a *RAS* mutation RFS of 7.1 months (95% CI 5.0–N/R months, *n* = 10) and with a *BRAF*^*V600E*^/*RAS* wild-type RFS of 9.4 months (95% CI: 7.2–N/R months, *n* = 13). Comparable outcomes were shown for patients with pMMR mCRC, demonstrating a median RFS of 7.2 months (95% CI: 4.8–11.5 months, *n* = 22), 7.0 months (95% CI: 6.3–7.8 months, *n* = 318), and 7.4 months (95% CI 6.6–9.4 months, *n* = 269), respectively.

Median OS for patients with dMMR mCRC was 33.3 months (95% CI 26.7–N/R months) compared with 43.5 months for patients with pMMR mCRC (95% CI 40.2–46.8 months), mainly due to poor survival of patients with dMMR mCRC in case of recurrence in the preimmunotherapy era. A total of 822 patients had recurrence of disease and additional follow-up was available for 98% (*n* = 39/40) for patients with dMMR tumors and 79% (*n* = 619/782) for patients with pMMR tumors. After recurrence, palliative systemic chemotherapy was administered in 56% of patients with dMMR tumors and 53% of patients with pMMR tumors. Three patients with dMMR tumors received immunotherapy after recurrence in a trial setting. Salvage local therapy occurred in 6 patients with dMMR mCRC (15%, *N* = 1 hepatectomy, *N* = 3 CRS ± HIPEC, *N* = 2 other) and 160 patients with pMMR mCRC (26%, *N* = 85 hepatectomy, *N* = 20 CRS ± HIPEC, *N* = 55 other). The difference in chance of salvage local treatment is mainly due to differences in metastasis pattern with more liver-only recurrences in patients with pMMR tumors (*N* = 213, 34%) compared with dMMR tumors (*N* = 3, 8%). In case of recurrence, a median OS of 10.2 months (95% CI 8.0–24.3 months) for dMMR mCRC and 27.4 months (95% CI 25.5–30.6 months) for pMMR mCRC was demonstrated (Supplementary Fig. S1).

### Uni- and Multivariable Analysis

Univariable analyses of ten preselected factors showed a significantly improved RFS for dMMR compared with pMMR (HR 0.62, 95% CI 0.44–0.88). Next to dMMR status, stage of disease (stage IV vs. stage I–III) was a significant predictor in univariable analysis (HR 1.22, 95% CI 1.04–1.43). Multivariable analysis demonstrated dMMR status as independent predictor for improved RFS (HR 0.57, 95% CI 0.38–0.87). Both stage IV disease (HR 1.24, 95% CI 1.03–1.49) and poor differentiation grade of primary tumor (HR 1.38, 95% CI 1.03–1.84) were predictive of a poor RFS (Table 2). Notably, perioperative systemic therapy, *BRAF* mutation status, sidedness, and type of local therapy were not univariable nor multivariable significant predictors.

**Table 2:** Univariable and multivariable regression of preselected factors on recurrence-free survival

Variable	Category		Univariable regression			Multivariable regression		
			HR^1^	95% CI^1^	*p*-value	HR^1^	95% CI^*1*^	*p*-value
Age	–	*–*	1.00	0.99, 1.00	0.4	1.00	0.99, 1.00	0.3
Sex	Female	(vs. male)	0.91	0.78, 1.06	0.2	0.88	0.75, 1.03	0.1
Sidedness	Right-sided	(vs. left-sided)	0.95	0.79, 1.14	0.6	0.98	0.80, 1.19	0.8
	Rectosigmoid	(vs. left-sided)	1.18	0.99, 1.40	0.1	1.18	0.98, 1.41	0.1
Differentiation grade	Poor	(vs. moderate/well)	1.16	0.90, 1.49	0.2	1.38	1.03, 1.84	**0.030**
Stage	Stage IV	(vs. stage I–III)	1.22	1.04, 1.43	**0.017**	1.24	1.03, 1.49	**0.024**
*BRAF*^*V600E*^ mutation status	Mutation	(vs. wildtype)	0.96	0.65, 1.43	0.9	1.41	0.82, 2.42	0.2
*RAS* mutation status	Mutation	(vs. wildtype)	1.11	0.93, 1.33	0.2	1.15	0.92, 1.42	0.2
Mismatch repair status	dMMR	(vs. pMMR)	0.62	0.44, 0.88	**0.007**	0.57	0.38, 0.87	**0.009**
Type of local treatment	CRS/HIPEC only	(vs. CRLM only)	0.84	0.69, 1.02	0.1	0.90	0.72, 1.12	0.3
Perioperative chemotherapy	Received	(vs. not received)	1.07	0.93, 1.24	0.3	0.91	0.77, 1.08	0.3

Bold-values are considered significant (*p*-value < 0.05)

*CI* confidence interval, *CRLM* colorectal liver metastasectomy, *CRS/HIPEC* cytoreductive surgery/hyperthermic intraperitoneal chemoperfusion, *dMMR* deficient mismatch repair, *HR* hazard ratio for RFS, *mCRC* metastatic colorectal cancer

## Discussion

We present survival outcomes of the current largest real-world data cohort of patients with dMMR mCRC and pMMR mCRC treated with local therapy in the preimmunotherapy era. Consensus of curative-intent local therapy of metastases was mainly based on studies including patients with pMMR mCRC while its benefit is not known for the distinctive biological subgroup of patients with dMMR mCRC.^3^ Our results show that median RFS is longer for patients with dMMR mCRC compared with pMMR mCRC with a median RFS of 11.1 months compared with 8.9 months. The proportion of patients who remain free of recurrence is larger in patients with dMMR mCRC; 2-year RFS was 43% in dMMR mCRC and 21% in pMMR mCRC. When correcting for important differences in characteristics between groups, such as sidedness of primary tumor, type of local therapy, and perioperative chemotherapy, dMMR remains a strong independent predictor for improved RFS compared with pMMR with a HR of 0.57 (95% CI 0.38–0.87, *p* = 0.009).

The median OS of patients with dMMR mCRC is shorter than for patients with pMMR mCRC (33.3 months compared with 43.5 months). This is likely due to reduced survival after disease recurrence for patients with dMMR mCRC, in the era before standard immunotherapy, with a median OS of 10.2 months compared with 27.4 months for patients with pMMR mCRC. This is in line with the lower response rates to systemic chemotherapy in patients with dMMR compared with pMMR mCRC and the generally more favorable prognosis of patients with pMMR mCRC with systemic chemotherapy in later lines of treatment.^6,18^ Additionally, salvage local therapy occurred more often in patients with pMMR mCRC tumors, potentially due to more frequent liver-only recurrence and possibilities for repeated hepatectomy. This likely contributes to the improved OS after first recurrence for patients with pMMR tumors. Notably, in our cohort, immunotherapy could not have affected median OS, since reimbursement of immunotherapy in The Netherlands is arranged since February 2021 and OS data were retrieved through the Municipal Personal Records Database on 31 January 2021.

As expected, we found that patients with dMMR tumors were less often treated with local therapy (22% dMMR vs. 47% pMMR). There are various causes why patients with dMMR mCRC may receive treatment with local therapy less often. Patients with dMMR mCRC present more frequently with metastatic sites involving lymph nodes, which is less often amenable for curative-intent local treatment compared with liver-only disease, which is often seen with pMMR mCRC.^7^ Furthermore, despite the subgroup of young patients with dMMR due to Lynch syndrome, in general, the dMMR population is older than the pMMR mCRC population, which could influence decision making regarding local treatment, especially regarding more extensive local treatment such as CRS ± HIPEC.^7^ In our real-world cohort, patients with dMMR mCRC had more tumors harboring *BRAF*^*V600E*^ mutations (50% vs. 4%), more right-sided tumors (78% vs. 27%), less liver-only disease (27% vs.70%), and more peritoneal involvement (44% vs. 18%), resulting in more CRS–HIPEC therapy (48% vs. 16%) and fewer CRLM resections (27% vs. 70%). The lower proportion of curative-intent local therapy of metastases in patients with dMMR mCRC is therefore probably caused by the distinct clinicopathological characteristics between the two groups. As immunotherapy was not the standard available in the inclusion period, this did not influence the decision making for patients with dMMR regarding local treatment and could not contribute to the lower proportion of patients receiving local therapy. Notably, nationwide, all patients receiving local treatment in this time period were included, limiting the risk of selection bias.

The absence of data concerning the tumor load of patients is a limitation of our study, e.g., peritoneal cancer index for CRS ± HIPEC or number of liver metastases for CRLM treatment, while this is recognized to be an important prognostic factor for recurrence.^19^ Lastly, to increase sample size, patients with dMMR mCRC were from the period 2015 to 2018 while patients with pMMR mCRC were from the period 2015 to 2016. However, we do not expect a better RFS or OS for patients with dMMR as a result of the more recent time period due to no availability of immunotherapy as standard of care at the time and no other important novel systemic treatments that became available in this period.

Scarce data exists for patients with dMMR mCRC treated with local therapy. Two exclusive CRS ± HIPEC studies included 15–44 patients with dMMR, both showing superior RFS and OS compared to pMMR.^15,20,21^ These results align with our study and show that local therapy of metastases in patients with dMMR mCRC that are not pretreated with immunotherapy can lead to durable RFS and reasonable OS. On the other hand, immunotherapy also shows durable responses and long-term survival benefit in patients with dMMR mCRC. This must be taken into account when determining the position of upfront local therapy in resectable patients with dMMR mCRC.^8^ Additionally, salvage local treatment following stable disease or partial response in patients treated with immunotherapy demonstrates a high 2-year RFS.^9–11^ A potential disadvantage of immunotherapy as first-line therapy in patients with dMMR mCRC with resectable disease is the chance of primary progression, which occurs in 30% of patients, potentially resulting in non-resectable disease afterwards.^8^ Additionally, 10% of patients treated with immunotherapy develop severe immune-related adverse events.^22^ A substantial subset of patients with dMMR in our study obtained a durable RFS and long OS, with a chance of cure by only local treatment, potentially sparing the treatment of immunotherapy. In clinical decision making, the (dis)advantages of both treatment options should be considered, including toxicity, risk of complications, and the possibilities regarding sequence of treatment.

## Conclusions

Patients with dMMR mCRC in the real world benefit from curative-intent local therapy of metastases in the preimmunotherapy era, with a median RFS of 11.1 months compared with 8.9 months for patients with pMMR mCRC. After 2 years, 43% of all dMMR mCRC were free of recurrence after local treatment compared with 21% of the patients with pMMR mCRC. Differences in metastatic pattern led to more CRLM resections in patients with pMMR mCRC and more CRS/HIPEC in patients with dMMR mCRC; however, when stratified for type of local therapy, a comparable to better RFS for dMMR mCRC is still demonstrated per treatment type. Furthermore, when correcting for important differences in patient characteristics, dMMR remains a significant independent predictor for improved RFS. The relatively large proportion of patients with dMMR mCRC who remain free of recurrence after curative-intent local treatment of metastases could aid in the decision of upfront immunotherapy versus local therapy in case of resectable metastases for patients with dMMR mCRC. This should be decided by shared decision making, weighing the chance of potential cure against the chance of adverse events from immunotherapy, and risk of complications of local treatment for every individual patient.

## Supplementary Information

Below is the link to the electronic supplementary material.

Supplementary file1 (DOCX 164 kb)

## Data Availability

J.R. and K.Z. had full access to all the data in the study and take responsibility for the integrity of the data and the accuracy of the data analysis.
