# Supplementary material for: Survival of Patients with Deficient Mismatch Repair Versus Proficient Mismatch Repair Metastatic Colorectal Cancer Receiving Curative-Intent Local Treatment of Metastases in a Nationwide Cohort
Source: Ann Surg Oncol. 2023 Aug 1;30(11):6762–70. doi: 10.1245/s10434-023-13974-7 (PMC10506947; doi:10.1245/s10434-023-13974-7)
Supplement: Supplementary file 1 — Supplementary file1 (DOCX 164 kb) [file 10434_2023_13974_MOESM1_ESM.docx]

**Supplements**

**Overall survival in patients after recurrence: dMMR *versus* pMMR mCRC**

**
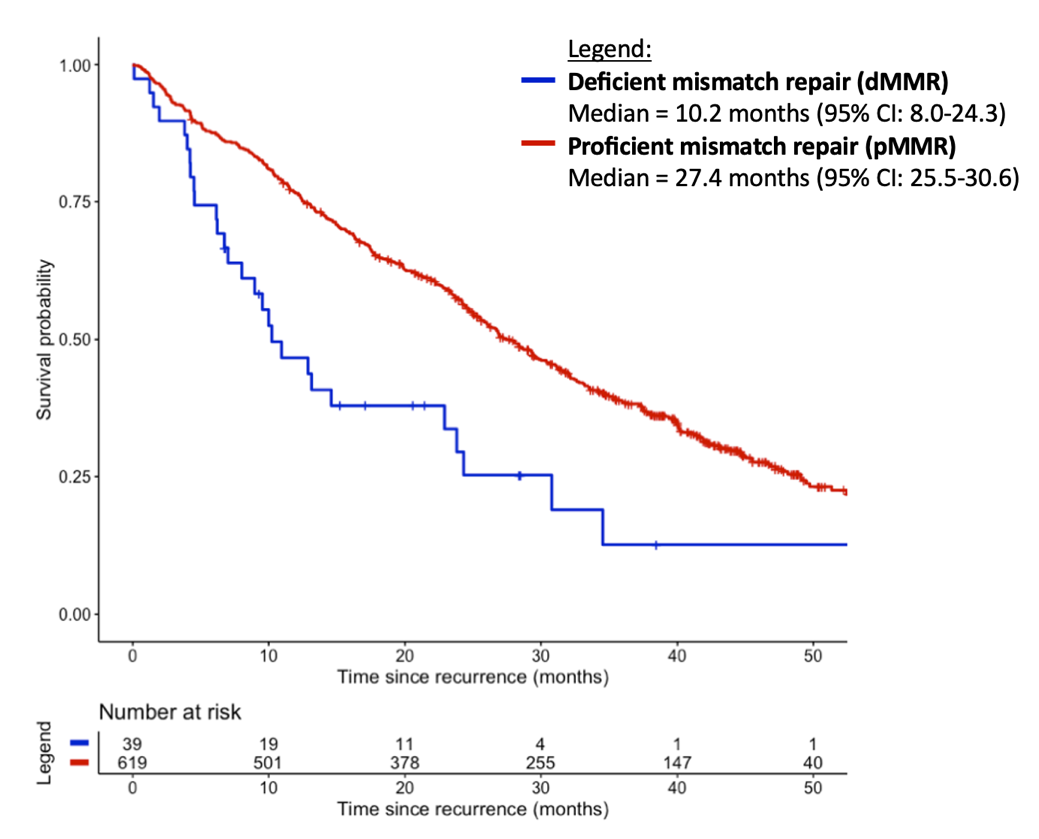
**

**Supplemental Figure 1.** Overall survival of deficient mismatch repair (dMMR) versus proficient mismatch repair (pMMR) metastatic colorectal cancer patients in patients after recurrence.
